# Health Risks, Preventive Behaviours and Respiratory Illnesses at the 2019 Arbaeen: Implications for COVID-19 and Other Pandemics

**DOI:** 10.3390/ijerph18063287

**Published:** 2021-03-22

**Authors:** Farah Al-Ansari, Masoud Mirzaei, Basma Al-Ansari, Mohammad Basim Al-Ansari, Mohammed Saeed Abdulzahra, Harunor Rashid, Grant A. Hill-Cawthorne, Mustafa Al Ansari, Joel Negin, Katherine Conigrave

**Affiliations:** 1 Faculty of Medicine and Health, Sydney School of Medicine (Central Clinical School), University of Sydney, Camperdown, NSW 2006, Australia; basma.al-ansari@sydney.edu.au (B.A.-A.); mustafa.alansari@sydney.edu.au (M.A.A.); kate.conigrave@sydney.edu.au (K.C.); 2 Yazd Cardiovascular Research Centre, Shahid Sadoughi University of Medical Sciences, Yazd 89151-73160, Iran; masoud_mirzaei@hotmail.com; 3 Office of Ayatollah Al-Ansari, Sydney, NSW 2200, Australia; mala8782@uni.sydney.edu.au; 4 Department of Medicine, College of Medicine, University of Kufa, Najaf 540011, Iraq; mohammed.alnaseri@uokufa.edu.iq; 5 The Children’s Hospital at Westmead, National Centre for Immunisation Research and Surveillance (NCIRS), Westmead, NSW 2145, Australia; harunor.rashid@health.nsw.gov.au; 6 The Discipline of Child and Adolescent Health, Children’s Hospital Westmead Clinical School, Faculty of Medicine and Health, University of Sydney, Westmead, NSW 2145, Australia; 7 Faculty of Medicine and Health, Sydney School of Public Health, University of Sydney, Camperdown, NSW 2006, Australia; grant.hill-cawthorne@sydney.edu.au (G.A.H.-C.); joel.negin@sydney.edu.au (J.N.); 8 Drug Health Services, Royal Prince Alfred Hospital, Missenden Rd, Camperdown, NSW 2050, Australia

**Keywords:** Arbaeen, global health, infectious disease transmission, public health, communicable diseases, mass gathering medicine, COVID-19, facemasks, handwashing

## Abstract

COVID-19 poses grave challenges for mass gatherings. One of the world’s largest annual gatherings, Arbaeen, occurs in Iraq. We studied respiratory symptoms and risk and protective factors using representative sampling of Arbaeen pilgrims in 2019 to inform prevention of COVID-19 transmission. Structured sampling was used to recruit walking pilgrims. A questionnaire asked about respiratory symptoms, risk, and preventive factors, including hygiene-related resources of toilet facilities. The commonest symptom reported by the 1842 participants (63.3% male, 36.7% female) was cough (25.6%). Eating in mawkibs (rest areas) with indoor kitchens and drinking only packaged water were associated with lower risk of cough (AOR = 0.72, CI = 0.56–0.94; AOR = 0.60; CI = 0.45–0.78, *p* < 0.05). Facemask use was associated with increased risk of cough (AOR = 2.71, CI = 2.08–3.53, *p* < 0.05). Handwashing was not protective against cough, or against (one or more of) cough, fever, or breathlessness in multivariate analysis. Toilet facilities often lacked running water (32.1%) and soap (26.1%), and had shared hand towels (17%). To reduce risk of respiratory infections including COVID-19 during Arbaeen or other mass gatherings, needs include running water, soap, and hygienic hand drying options or hand sanitiser. Education on proper handwashing and facemask approaches and monitoring around food preparation and eating spaces are needed.

## 1. Introduction

Mass gatherings have been held over history for sporting, social, religious, and other events. Due to the close contact of participants in congested areas, such gatherings have a high potential to accelerate transmission of infectious disease. A mass gathering of two million people in Mexico, the Iztapalapa Passion Play in April 2009, is believed to have accelerated the spread of the last pandemic caused by influenza A(H1N1)pdm09 [1]. Similarly, the spread of severe acute respiratory syndrome coronavirus 2 (SARS-CoV2) is thought to have been accelerated by the 2019 Spring Festival in China, and the 2020 Carnival in Brazil. These international mass gatherings coincided with the first known spread of the virus in China and the first reported cases in Latin America and Italy, respectively [2,3]. Pilgrims returning from religious mass gatherings in Iran are also thought to have carried the infection to Pakistan [4]. Mass gathering-related cases of COVID-19 have also been reported in South East Asian countries like Malaysia [5] and South Korea [6].

**Figure 1:**
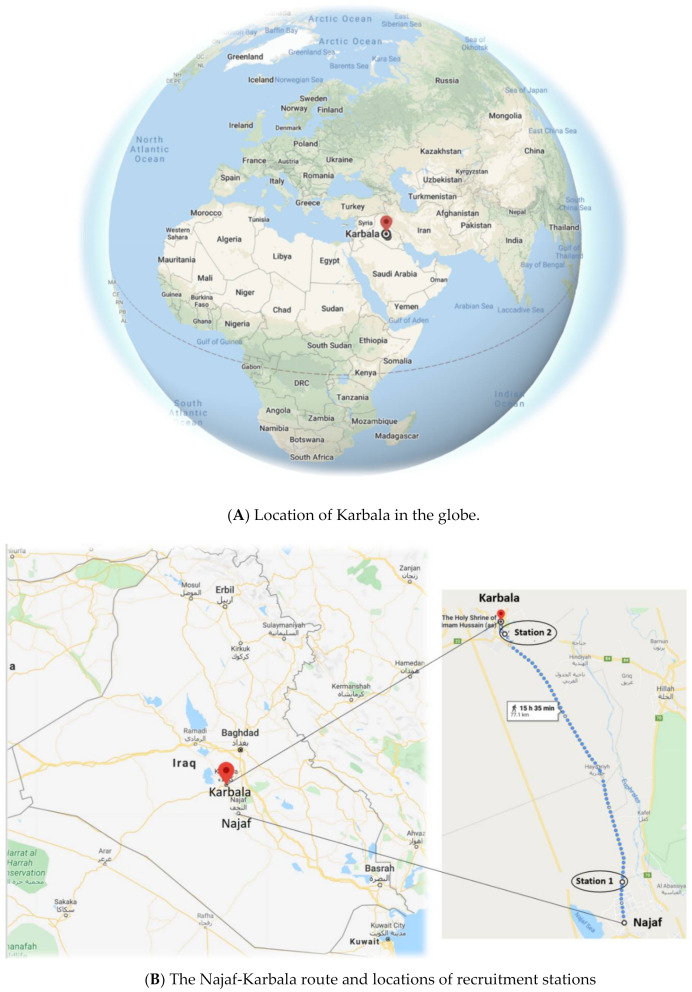
Karbala and its location in the globe (**A**) and in Iraq (**B**). Recruitment stations are shown in Figure 1B. Map adapted from Google Directions (Google Maps, 2020. Karbala, Iraq. Google Maps [online] Available through: https://www.google.com.au/maps/place/Karbala [accessed on 2 June 2020]) and checked by authors familiar with the region.

The Arbaeen walk in Iraq has an estimated 17–21 million participants [7,8,9], making it one of the world’s largest annual mass gatherings. Participants walk from cities across Iraq and from neighbouring countries to Karbala, a city in central Iraq [10] (Figure 1). The event commemorates the return of Hussein Ibn Ali’s family to the city 40 days after the massacre of Hussein and his relatives and companions by the then Caliph in 680 AD. Arbaeen occurs on every 20th day of the years’ second Islamic lunar calendar month, Safar. Use of the lunar calendar means that the Arbaeen event moves to different seasons over the years. This may affect the type of pathogens and possibly their transmission. The 2019 Arbaeen happened during summer in hot temperatures of up to 40 °C.

There is no specific starting point in Arbaeen. Participants head to Karbala from cities across Iraq and Iran. A number of participants also walk from other countries such as Turkey and India [10]. The main destination is the shrine of Imam Hussein in Karbala. However, some participants walk for shorter distances. In fact, some may even walk only a few steps towards Karbala just to take part in the pilgrimage. The three main routes to Karbala extend 80, 47, and 102 kms, from Najaf, Hilla, and Baghdad, respectively [10]. These routes are crowded with walkers for up to 10 days. The most popular and congested of these is the Najaf-Karbala route (Figure 1B) [10].

Arbaeen participants are provided with free services including accommodation, food, and drinks by volunteers along the walking routes [10]. Overnight stops and eating areas are clustered along the routes. The type of accommodation and food source could affect the risk of disease transmission. For example, many pilgrims stay in mawkibs, which are temporary or built structures designed to provide free accommodation and food preparation areas to pilgrims. Often, multiple pilgrims sleep on mats on the floor, and there may not be sufficient space for 1.5 m separation between groups. Bedding and plates are often shared, and in some, there is limited circulation of fresh air in enclosed spaces.

Arbaeen participants may believe their health risks are limited because of the religious focus of the walk [11]. For example, during the COVID-19 pandemic in 2020, the Arbaeen event did occur, though with fewer numbers. The number of 2020 Arbaeen participants entering Karbala was still reported to be just over 14 million [12].

Respiratory disorders were reported as one of the top three causes of death in health care facilities in governances south of Karbala in 2014 [13], after cardiovascular disease and injuries. In keeping with this, 40% of patients visiting health outlets along the Najaf-Karbala route in 2016 presented with fever and cough [14]. Similarly, respiratory symptoms were the most common symptoms reported by 191 participants interviewed during the walk in the 2017 Arbaeen [10]. However, that pilot study was small and used convenience sampling.

Accordingly, this study aims to investigate respiratory symptoms and associated risk and protective factors in walking pilgrims during the 2019 Arbaeen, with recruitment designed to give a larger and representative sample.

The study was conducted eight weeks prior to the first documented COVID-19 case internationally and the subsequent temporary ban of many mass gatherings internationally. An understanding of the health aspects of this mass gathering can aid in planning appropriate public health measures for future mass gatherings and for international travel more broadly.

## 2. Materials and Methods

### 2.1. Overview of the Study

This cross-sectional survey explored health risks, preventive behaviours, and illnesses of a sample of 1842 Arbaeen participants (aged 16 years and over) in Iraq in 2019. Recruitment was designed to be representative.

### 2.2. The Survey

A structured questionnaire was developed with 23 key items, including demographics, type of accommodation (household, outdoor mawkib, indoor mawkib, hotel, mosque/hussaynia and/or other), and food services used. Outdoor mawkibs are tents set up during Arbaeen by volunteers to provide accommodation and food. Indoor mawkibs are permanent structures built to accommodate pilgrims, mainly during the Arbaeen.

The survey asked about a range of preventive measures used by participants including use of facemasks, handwashing, avoidance of street food, use of only packaged water (bottled or sealed packaged cups), and avoidance of fresh fruits and salads (tick boxes used). We also asked about the hygiene-related resources of toilet facilities at the participant’s last place of accommodation (running water, soap availability, shared towel).

Participants were asked whether they experienced the following symptoms: cough, breathlessness, sore throat, fever or influenza-like illness (combination of cough, sore throat, and fever). Participants were asked about pre-existing conditions (lung problems including asthma, heart problems, diabetes, kidney disease) and whether they were up to date with vaccinations, had an influenza vaccination in the past year, and had received (other) travel-specific vaccinations. Usual smoking status was enquired into (see questionnaire in Appendix A).

### 2.3. Survey Administration

Study data were collected and managed using REDCap electronic data capture tools hosted at the University of Sydney [15,16]. The survey was provided in five languages (English, Arabic, Farsi, French, and Urdu). It was administered on mobile phones or tablet computers provided by the interviewers. The survey could be completed offline.

### 2.4. Interviewers

Volunteer interviewers/research assistants [RAs] (*n* = 42; including three of the authors [FA, BA, MBA]) collected the data. This included interviewers who spoke English, Arabic, Farsi and/or French (*n* = 33, 32, 9, and 5, respectively). None of the interviewers spoke Urdu. All the Arabic-speaking interviewers, except three of the authors and one dentist, were Iraqi medical students. Six of the Farsi-speaking interviewers usually work as professional interviewers in Iran. Two workshops were conducted to train RAs on recruitment and data collection.

### 2.5. Recruitment

Recruitment occurred over 3 days (15–17 October 2019, 2–4 days before Arbaeen) spread over 5–8 h per day, excluding a 2–3 h daily break. These days were chosen as most people walk between Najaf and Karbala in the final days of the Arbaeen. We did not recruit on the day of Arbaeen and the preceding day as pilgrims are considered likely to be occupied with the mourning associated with these days, and to be keen to continue walking to complete their pilgrimage.

Two recruitment stations were set up, one on the outskirts of Najaf and the other close to Karbala (Figure 1B). Each station consisted of a tent with chairs.

Recruitment at Station 1, and on the final day at Station 2 occurred during the morning and afternoon. Recruitment at Station 2 was conducted during the afternoon and evening on the first day, and morning, afternoon, and evening on the second day.

At each station, a RA acting as a ‘counter’ selected every 15th person passing an allocated spot. At the end of day 2, the percentage of women in our sample was 30%, despite a relatively equal male-to-female ratio observed amongst walkers. Accordingly, for 3 h on day 3 we over-recruited women, selecting the first woman from the 15th person onwards.

The counter directed an interviewer, whose language was likely to match the language of the 15th passer-by, to approach that person. For example, if the participant appeared to be Iranian based on appearance or clothing, a Persian-speaking interviewer approached them. Where the participant was assumed to be more comfortable being interviewed by someone of the same gender (e.g., ladies wearing niqab) the same gender interviewer approached.

It was made clear that participation was voluntary. If the participant did not speak the language of the RA, the RA showed a card with study information and an invitation to participate in the five languages.

If the person declined, their gender, estimated age group, and reason for refusal (stated or observed) were recorded. Where the participant consented, the interviewer read out the survey questions from the App, and recorded responses. If the interviewer did not speak the language of the participant, participants filled out the electronic form themselves.

### 2.6. Analysis

Country of origin was coded according to the country’s income level, using the World Bank classification [17]. Statistical analysis was conducted using SPSS version 24. Differences in the prevalence of symptoms, preventive measures used, food source and other parameters were explored using descriptive bivariate analysis. Multivariate logistic regression was used to examine independent predictors of the development of cough. Age, gender, and any other factors that were associated with development of cough in bivariate analysis were entered as predictors. Where two predictors were closely correlated, only one was entered into the equation. A similar analysis was done for prediction of any one of cough, fever, and breathlessness.

### 2.7. Ethics

Ethics approval for the study was obtained from Kufa University, Iraq (3 September 2019).

## 3. Results

### 3.1. Demographics

Of the 2704 individuals approached, 1842 (67.9%) agreed to participate (63.3% male, 36.7% female; gender not recorded for four individuals; Table 1). Males invited to the study were more likely to accept participation than females (71.0% vs. 63.7%, respectively). Also, younger individuals were more likely to accept than older pilgrims (consent rate of 72.4%, 66.9%, 47.6% for reported or estimated age groups of <35, 35–60, and >60 years, respectively). The majority of the sample were either Iraqi (43.1%; *n* = 782) or Iranian (51.5%, *n* = 933). The remainder (*n* = 98, 5.4%) were from Pakistan (*n* = 32), Saudi Arabia (*n* = 15), Lebanon (*n* = 11), Kuwait (*n* = 9), Bahrain (*n* = 8), India (*n* = 5), and three or fewer each from Afghanistan, Syria, Turkey, Australia; Azerbaijan, United Kingdom and United States of America. The survey was most commonly completed in Persian or Arabic (49.5% and 48.3% respectively), with only a small percentage using English (*n* = 25, 1.4%) or Urdu (*n* = 17, 0.9%). No participants completed the survey in French. The most common age group of participants was 25–44 years (*n* = 839, 46.3%, Table 1). Only 54 participants (3.0%) were aged 65 and over. A total 23 participants did not provide their age.

The most common accommodation reported was outdoor/tent mawkibs (41.9%; Table 1). This was followed by private houses, mosque/hussaynia and built/permanent mawkibs (30.0, 28.9, and 22.2%, respectively). Hotels were used by only 3.1% of participants. The most common food source was mawkibs (with outside cooking facilities, 60.4%; with indoor kitchen facility, 51.3%), followed by donated street food (44.7%). Food from private homes, commercial street sales, and restaurants were less common (12.6%, 5.4%, and 2.0%, respectively; Table 1). Participants could nominate more than one source.

### 3.2. Reported Symptoms

The most prevalent symptom was cough (*n* = 461; 25.6%; with 11.4% productive and 13.6% dry; Table 2), followed by sore throat, then breathlessness and fever (reported by 18.0%, 12.1%, and 8.2%, respectively). The combination of fever, sore throat, and cough was reported by only 58 (3.2%) participants. Of the 461 participants who reported cough, the majority (65.5%) reported its duration as two or fewer days, while just under a quarter (24.2%) reported 3–7 days. In keeping with this, most (*n* = 405, 87.8%) developed their cough after starting their walk. Similarly, most participants who reported breathlessness or fever (83.0% and 95.2%, respectively) developed the symptoms after starting their walk. Onset of sore throat was not asked due to time constraints. Further analysis for sore throat was therefore not conducted.

Participants who had been walking for 6 days or more were more than twice as likely to report at least one symptom of cough, breathlessness, or fever than those who had been walking a shorter time (72.9% vs. 29.0%, respectively, *p* < 0.001, Table 3).

A higher percentage of young participants (aged 16–24) developed any one of cough, breathlessness, or fever during the walk than older people (38.9%, *p* = 0.002, Table 3). The prevalence of experiencing any of these symptoms steadily fell with increasing age, to 22.6% of those aged 65+ years.

Participants from the combination of low to lower-middle income countries and those from upper-middle income countries were more likely to experience cough than the 37 participants from high-income countries (28.2 and 23.1% vs. 5.4%, respectively, *p* = 0.024).

### 3.3. Preventive Measures/Risk Factors and Association with Infectious Disease Symptoms

**Figure 2:**
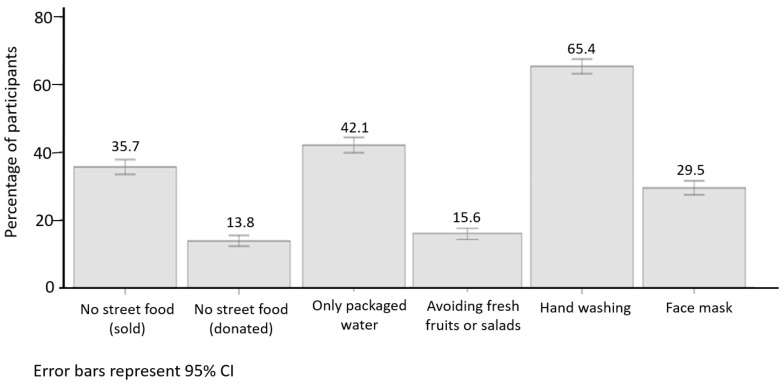
Prevalence of health-related preventive measures used by 1842 Arbaeen participants in 2019.

The most common preventive measure used by Arbaeen participants was handwashing, reported by 65.4%. This was followed by the use of only packaged water (bottled or sealed packaged cups; 42.1%), no commercial street food (35.7%), and a facemask (29.5%). A smaller percentage of participants avoided fresh fruits or salads and/or donated street food (15.6 and 13.8%, respectively, Figure 2).

**Figure 3:**
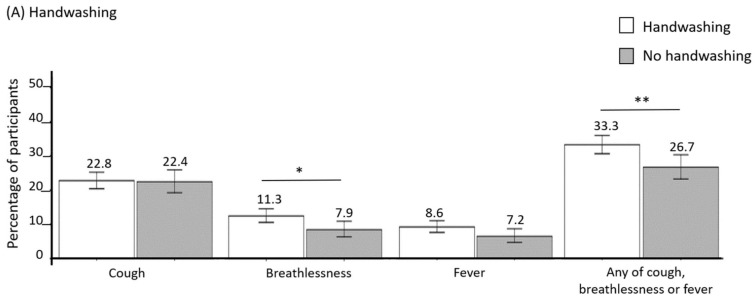

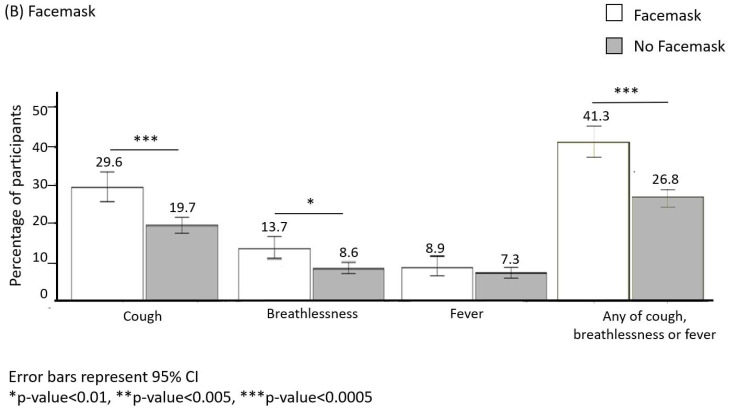
Comparison of the prevalence of respiratory symptoms that started during the walk in participants who used handwashing (**A**) or facemask (**B**) as preventive measures to that of those not using those measures in a sample of 1842 Arbaeen participants in the year 2019.

Both handwashing and facemask use were associated with increased risk of key symptoms on bivariate analysis. Experience of any of breathlessness, cough or fever, and of breathlessness, in particular, was significantly more common in those who reported handwashing as a preventive measure compared with those who did not use that measure (33.3 vs. 26.7%, and 11.3 vs. 7.9%, *p* = 0.005 and 0.026, respectively, Table 3, Figure 3A). Facemask use was associated with a significantly higher risk of developing cough and/or breathlessness (29.6 vs. 19.7%, 13.7 vs. 8.6%, *p* = 0.000 and *p* = 0.001, respectively, Table 3, Figure 3B) compared with individuals who did not use that measure.

The use of only packaged water was associated with a lower risk of developing any one of cough, breathlessness, or fever, and a lower risk of developing cough (25.8 vs. 35.0%; 16.7 vs. 27.1%, respectively, *p* < 0.001, Table 3). Similarly, participants who avoided fresh fruits and salad were less likely to develop any one of breathlessness, cough or fever, and cough and fever in particular (25.3 vs. 32.2%, 14.5 vs. 24.2% and 3.8 vs. 8.5%, *p* = 0.022, *p* < 0.001 and *p* = 0.007, respectively, *p* = 0.007).

Being a regular smoker was a risk factor for development of both cough (26.3% vs. 21.2%, *p* = 0.025) and breathlessness (14.9% vs. 9.1%, *p* = 0.002, Table 3) compared with those who were not regular smokers.

The majority of participants (60.9%) reported being up to date with their vaccinations (details not obtained). One in six (16.0%) participants reported influenza vaccination in the past year, but only 5.0% (*n* = 86) had other vaccinations for this trip (Table 3). Being up to date with vaccinations and/or vaccinated against influenza was not associated with lower incidence of one or more of the respiratory symptoms investigated. Having had other vaccinations for the trip was associated with a lower risk of developing breathlessness (3.4 vs. 10.6%, *p* = 0.032) but a higher risk of fever (21.8 vs. 7.0%, *p* < 0.001).

### 3.4. Pre-Existing Conditions

Almost a third of participants (*n* = 569; 30.9%; 25.1% of males and 41.0% of females) reported pre-existing conditions. The most common was hypertension (8.9%). Low percentages of participants (3% for each) reported lung problems (including asthma), heart problems, diabetes, or kidney disease. Eight percent reported ‘other’ conditions. Participants with pre-existing lung problems (including asthma) had nearly twice the risk of developing at least one of cough, breathlessness, or fever (59.6% vs. 30.1%, *p* < 0.015) compared to others.

### 3.5. Toilet Facilities

Most (95.1%) participants reported having toilet facilities at their last night’s accommodation. Of these, one third (*n* = 595, 32.4%) said they had no running water for handwashing; and over a quarter (*n* = 481, 26.1%) reported no soap or detergent for handwashing (absent for 15.1% males vs. 10.9% females, *p* < 0.05). Approximately 17% of participants reported a shared towel for drying hands.

### 3.6. Multivariate Predictors of the Development of Cough and at Least One of Cough, Breathlessness, or Fever

Age, gender, and factors significantly associated in bivariate analysis with cough and (separately) with experiencing at least one of cough, breathlessness, or fever were examined in logistic regression for these outcomes (Table 4). Since recruitment site (station one vs. station 2) was significantly associated with the walking duration, only walking duration was included. Similarly, because of the inverse association between eating donated street food and avoiding donated street food, only eating donated street food was included. As flu vaccination findings were only significant on bivariate analysis when ‘unknowns’ were included in the comparator group, this was not included.

Increasing age was significantly associated with a marginal decrease in risk of cough (OR = 0.99, 95% CI = 0.98–1.00) on multivariate analysis.

Individuals who had walked for 6 or more days were 4.6 times more likely to develop a cough (AOR 4.59, 95% CI = 3.01–7.00) and 7.2 times more likely to develop one of the respiratory symptoms (AOR 7.20, 95% CI = 4.64–11.20, Table 4) on multivariate analysis.

Those eating in mawkibs with indoor kitchen facilities, eating donated street food, avoiding fresh fruits and salads, and drinking only packaged water were less likely to develop cough (adjusted OR [AOR] 0.72, 95% CI: 0.56–0.94; 0.66, 0.51–0.85; 0.58, 0.39–0.86; 0.60, 0.45–0.78). Similarly, using drinking packaged water, and eating donated street food was associated with a reduced risk of one or more of the three respiratory symptoms (AOR = 0.64, 95% CI = 0.50–0.81; 0.76, 95% CI = 0.61–0.94).

In keeping with these findings for cough, risk of at one or more of these respiratory symptoms was higher in those who ate food from an outside cooking mawkib (AOR = 1.36, 95% CI = 1.08–1.72) than those who did not. Risk was also higher in those who avoided sold street food (AOR = 1.37, 95% CI = 1.09–1.72) compared with those who did not.

Those using facemasks were 2.6 times more likely to develop cough (AOR 2.59; 95% CI: 1.99–3.37) and 2.5 times as likely to develop one of the three respiratory symptoms (AOR 2.47; 95% CI = 1.95–3.13, *p* < 0.001,) Table 4).

For each dependent variable, independent variables examined were age, gender, and any predictors that were significant in bivariate analysis for that outcome.

For cough, independent variables examined are: gender, age, walk duration, type of country of origin (high income vs. others), mawkib food (outside cooking), mawkib food (kitchen facility), eating donated street food, regular smoking, use of face mask, use of only packaged water (bottled or packaged cups) and avoiding fresh fruits and salads.

For any of the three symptoms (cough, breathlessness, or fever) independent variables examined were: gender, age, walk duration, mawkib food (outside cooking), use of face mask, use of handwashing, avoiding fresh fruits or salads, avoiding commercial street food, eating donated street food, and use of packaged water (bottled or packaged cups).

## 4. Discussion

### 4.1. Results Discussion

The current study is the first to have attempted to recruit a representative sample of Arbaeen walkers to examine respiratory symptoms, risk, and preventive factors. It demonstrated that respiratory symptoms were common among walkers, with cough affecting over a quarter (25.6%) and sore throat affecting 18.0%. It also showed breathlessness and fever prevalence to be 12.1% and 8.2%, respectively. Neither reports of handwashing nor of mask wearing were associated with a reduction in the development of respiratory symptoms. Indeed, mask wearing was associated with an increased risk of respiratory symptoms on multivariate analysis. Though we do not know if mask wearing preceded or followed development of symptoms.

The relatively high prevalence of cough is in keeping with the 2017 Arbaeen pilot study [10]. Similarly, among patients visiting health outlets along the Najaf-Karbala route during the 2016 Arbaeen, 40% presented with fever and cough [14]. In contrast, in Wassit governance (on the eastern route to Karbala), only 2% of patients visiting mobile clinics presented with fever and cough during the 2014 Arbaeen [18]. It is not clear why the figure was so low. In the period 3–4 months after the 2013 Arbaeen, acute respiratory infections were reported by nearly half (48%) of pilgrims attending Iranian-specific clinics in Iraq [19]. In general, there is a lot of dust in the air during hot dry weather in Iraq. The roads are unsealed, and passing cars and pedestrians raise dust into the air. This perhaps also contributes to coughing.

Cough is also a very symptom during the Haj [20,21,22,23,24], where it is at least twice as common (50–93%) as reported here for the Arbaeen [25]. In the current study, the prevalence of influenza-like illness (combination of fever, cough, and sore throat; 7.8%) was a tenth of that found in Haj (78.2%) [26], and sore throat (18%) less than one sixth (82%) [27]. A key reason for these differences could be that most Haj studies involve surveys or cohort follow-ups conducted after the pilgrimage. In contrast, in the current study, over half the participants were in the first three days of their walk. They would have had less time for exposure and incubation of a respiratory infection. In keeping with that, in the current study, participants who had walked for 6+ days were more than seven times as likely to report at least one symptom of cough, fever or breathlessness than those walking for shorter times (AOR = 7.20, 95% CI = 4.64–11.22, *p* < 0.001).

Arbaeen participants are also generally younger than Haj participants, and so may be less susceptible to illness [10,28]. Though, during the COVID-19 pandemic, younger adults have sometimes been more prone to infection [29], perhaps because they take fewer precautions. In this study, cough was indeed more prevalent in those aged 16–24 years. After controlling for precautions taken and gender, older age was associated with only a marginal reduction in the risk of cough (OR = 0.99, 95% CI = 0.98–1.00) and was not a predictor of the three respiratory symptoms.

In the current study, the odds of developing cough was more than double (AOR 2.71; 95% CI = 2.08–3.53) in participants using facemasks compared to those not using them. A recent systematic review suggests that facemask use reduces transmission of SARS-CoV-1, MERS-CoV, and SARS-CoV-2 [30]. We do not have data on the temporal association between mask use and symptoms, so cannot exclude the possibility that some individuals started wearing the mask after they developed a cough, to protect others. Also, those more at risk of respiratory symptoms may be more likely to wear a mask. Onsite investigators also noted that the nature and quality of facemasks used, and their placement on the face varied widely.

Facemasks are recommended in crowded places, including during the Haj [31]. However, evidence for the effectiveness of facemasks during Haj is variable [32]. While a systematic review showed facemask use to be generally effective against clinically evident respiratory infections among Haj pilgrims (relative risk [RR] = 0.89, 95% CI: 0.84–0.94) [33], study endpoints varied widely. The one study which used laboratory-proven viral infections as an endpoint, did not show masks to be protective [33,34]. A study on medical mission personnel in Haj, showed that continuous use of a facemask was not associated with lower risk of respiratory symptoms, and intermittent use more than doubled the risk [35]. A recent observational study conducted at Haj over four consecutive years (2014 to 2017) found pilgrims who reported using facemasks had a higher likelihood of influenza-like symptoms (adjusted RR, 1.42; 95% CI, 1.10 to 1.82) and of acquiring rhinovirus infection (adjusted RR, 1.30; 95% CI, 1.03 to 1.65) [36].

Our findings of an increased risk of respiratory symptoms among individuals who used a facemask also raises questions around the quality of masks or mask use by Arbaeen participants. Several user factors can render masks less effective: incorrect wearing of the mask [37]; touching its outside then touching the nose [35]; touching the inside of the mask with unwashed hands; direct and indirect contact of the facemask with contaminated surfaces, lack of washing of a re-useable facemask between uses; prolonged use or reuse of masks designed as disposable; or even sharing of a mask. It is not clear to what extent these occur during Arbaeen. In addition, when facemasks become wet, they may increase the transmission of infection [38]. Furthermore, respiratory pathogens can have multiple routes of transmission, which may include via surfaces or objects that have been contaminated with the virus. The number of people who attend Arbaeen and their close proximity within and between accommodation and eating facilities may be a factor contributing to the lack of a protective effect for facemasks.

In general, our observations and those from Haj [36] and the contrasting evidence from the recent COVID-19 related literature review [30] suggest a need for caution in relying on facemask use for protection, the need for further research, and the need for user education about improved facemask use in the context of mass gatherings.

While handwashing also was associated with an increased risk of developing at least one of the three respiratory symptoms investigated during the Arbaeen in bivariate analysis, it did not remain significant in multivariate analysis. An observational study conducted at the Haj 2012 found that the overall prevalence of respiratory viruses during Haj was higher in pilgrims who reported more frequent handwashing than usual compared to those who practiced usual handwashing (53.6% vs. 23.3%; *p* = 0.018). The investigators proposed that this was most likely due to ‘reverse causation’, meaning ill pilgrims may have washed their hands more frequently [39]. Another possible explanation is that handwashing may not have been optimal. The latter would be supported by our finding that more than one third of respondents reported that the toilet facilities at their last night’s accommodation did not have running water (32.1%), and many lacked soap (26%) or had a shared hand towel (17%). Touching of buckets, taps, or other surfaces while washing hands may have increased risk of disease transmission. It is also common to see Arbaeen participants drying their hands with their own clothes or scarves. These items of clothing may have become contaminated.

We did not enquire into the use of hand sanitisers, because of time constraints. Use of alcohol-based hand sanitiser has been shown to reduce the risk of acute respiratory infections in Haj [35,40]. However, recent scientific discussion suggests that in real life scenarios, there is limited likelihood of respiratory infection (such as COVID-19) transmission via touching surfaces [41].

Interestingly, drinking only (sealed) packaged water was associated with a lower risk of cough and any of cough, fever, or breathlessness. Arbaeen walk participants can reach into big buckets of iced water to take sealed packages or bottles of water as they pass by. As viruses (including coronaviruses) can survive well at low temperatures [42,43], there have been concerns that this practice may cause disease transmission. However, the sealed water containers may convey less risk than available alternatives, such as water from a reuseable cup.

As pathogens can survive on surfaces, food preparation and handling can be a potential route of transmission. Eating food from mawkibs with an indoor kitchen and eating donated street food was associated with a lower risk of cough (AOR = 0.72, 95% CI = 0.56–0.94 and AOR = 0.66, 95% CI = 0.51–0.85, respectively) than not doing so. This may reflect the fact that in mawkibs with kitchen facilities a specific group of people prepare the food. Similarly, donated street food is mostly prepared by more private, indoor kitchens. In contrast, eating food from an outside cooking facility at a mawkib was associated with a higher risk of developing any of cough, breathlessness, or fever (AOR = 1.33, 95% CI = 1.08–1.72). With outside cooking facilities, walkers may come and join in food preparation. Their standards of food hygiene may vary. Also, plates, cutlery, and food preparation surfaces in outside areas can be exposed to aerosols or contact from passers-by or may not be washed well with detergent and running water.

Perhaps surprisingly, eating food from a private home was associated with increased risk of fever (OR = 1.80, 95% CI = 1.10–2.96) than not doing so. This association remained significant on multivariate analysis (data not shown). Many private homes on the route to Karbala are opened to pilgrims. They are often small in size, so congestion is common. Interestingly, participants who had eaten at a restaurant were five times as likely to develop fever (OR = 4.94, 95% CI = 1.93–12.62) than others in multivariate analysis (data not shown). In keeping with this, in the 2017 Arbaeen pilot study [10] and another study from Haj [44] eating at a restaurant was associated with a higher risk of diarrhoea. However, gastrointestinal illness does not seem to explain the fever in the current study, as eating at a restaurant was not associated with diarrhoea or vomiting (data not shown). Imperfect hygiene of restaurants (e.g., lack of adequate cleaning of chairs, tables between customers or even plates and cutleries) during Arbaeen could contribute to transmission of respiratory infections.

The current sample had a small percentage of participants (16%) vaccinated against influenza, and this vaccination was not associated with a lower risk of respiratory symptoms. Similarly, findings on influenza vaccination from Haj are mixed. One study suggested that influenza vaccination does not reduce risk of respiratory symptoms in Haj pilgrims [45], though two other studies demonstrated its effectiveness [46,47]. This requires further research [48]. It also raises concerns of reliance on the newly developed COVID-19 vaccinations in controlling the spread of the disease in mass gatherings.

In the current study, neither male gender nor smoking status remained significant predictors of cough development on multivariate analysis. Similarly, there was no difference in the prevalence of acute respiratory infection among male and female pilgrims in Haj [49]. Similarly, gender does not appear to increase the risk of contracting COVID-19, [50]. Also, although smoking can increase the risk of developing some respiratory tract infections, including influenza [51], there is no current evidence for an increased risk of acquiring COVID-19 [52].

### 4.2. Limitations

Despite efforts to obtain a representative sample of walking Arbaeen participants, in the current study, as in other Arbaeen studies [10,53], women seem to have been under-recruited (36.6% participated vs. 50% observed). Also, younger individuals were more likely to agree to participate. Also, although our survey was in five different languages, it was observed that some walkers could not participate due to a language barrier (*n* = 122, 14.2% of those who refused participation). Some participants may be illiterate or have visual problems so may not have been able to read the invitation card or screen-based survey. The small numbers of participants completing the survey in Urdu, and none completing it in French, could reflect either lack of interviewers who spoke those languages, or that participants who spoke those languages also spoke another language on the App. It is also possible that people from countries that spoke those languages were underrepresented along this Arbaeen route. Future studies could include interviewers who speak other common languages of the pilgrims, including Turkish, and additional survey translations.

While it is unlikely, we cannot exclude the possibility that the same person participated in the survey at both recruitment stations (1 and 2).

An important limitation to consider is the time required for infections to be acquired or for symptoms to appear. Some individuals may not have been walking long enough to be exposed and develop symptoms. We did not enquire into duration of sore throat. So, it is possible that some participants had this symptom before the walk started.

The questionnaire used was adapted from the one used for the 2017 pilot study [10] but has not been formally validated. The use of multiple statistical comparisons can increase the risk of type I error. However, key findings, such as the increased symptoms among individuals who used facemasks, remain significant when a Bonferroni correction is applied.

As we did not collect data on when facemask use started, we cannot exclude reverse causation in relation to the association between facemask use and respiratory symptoms.

Although interviewers were intensively trained, most were medical students rather than professional research assistants.

### 4.3. Implications of This Study

Standard protective measures like facemasks and handwashing are dependent on the available resources, and the way these are used. Education on wearing and storage of facemasks is important to avoid undermining their value, or even increasing risk of infection. Similarly, education around the need for soap or detergent when handwashing and the risks of shared towels or using own clothes for hand drying is required. Disposable hand towels would also reduce the risks.

It is a challenge to increase availability of running water, soap and safe hand drying equipment for large crowds for mass gatherings in any country. The challenge is greater in low or middle-income countries. However, regular disinfection of taps and other surfaces in eating areas, and other areas where people gather, and in toilet facilities is likely to reduce disease transmission. Increasing the availability of alcohol-based hand sanitisers is likely to be beneficial. Implementation of a range of prevention strategies is likely to help limit disease transmission at this and similar mass gatherings. Efforts at social distancing remain important.

## 5. Conclusions

Preparedness for a mass gathering event such as the Arbaeen is vital to limit infectious disease transmission [54]. This can help protect participants and the communities and countries that they return to. Lessons learned from this study may help reduce risks of transmission of respiratory viruses, including influenza and SARS-CoV-2.

Standard advice on precautions such as use of facemasks, handwashing, and social distancing, all need tailoring for the mass gathering setting and for the developing world. Social distancing is very difficult during such a gathering but remains important. Attempting to space out people in locations where they gather to eat could be helpful. Pilgrims can also be encouraged to select less crowded eating areas where possible. It may also be necessary to introduce education on, and oversight of, food preparation hygiene during the Arbaeen. Hand sanitiser is likely to be particularly important where there is a shortage of running water.

These lessons will also be useful in controlling disease transmission during regular social activities and travel in the developing world.

## Figures and Tables

**Table 1:** Demographics of Arbaeen 2019 survey participants for whom data were available.

	Male *n* = 1163		Female *n* = 675		*p*-Value	Total *n* = 1838 ^a^	
	*n*	(%)	*n*	(%)		*n* ^a^	(%)
**Age (years)**					<0.001		
16–24	**309**	**(26.6)**	**128**	**(19.0)**		438	(24.1)
25–44	**556**	**(47.8)**	**283**	**(41.9)**		841	(46.2)
45–64	**247**	**(21.2)**	**239**	**(35.4)**		487	(26.7)
65+	**41**	**(3.5)**	**13**	**(1.9)**		54	(3.0)
**Country of origin**					<0.001		
Iraq	**426**	**(36.6)**	**356**	**(52.8)**		782	(42.6)
Iran	**667**	**(57.4)**	**267**	**(39.6)**		933	(50.8)
Low to lower middle income	**30**	**(2.6)**	**13**	**(1.9)**		43	(2.3)
Other upper middle income	**3**	**(0.3)**	**12**	**(1.8)**		15	(0.8)
High income	**24**	**(2.1)**	**13**	**(1.9)**		37	(2.0)
Unknown (non-recorded)	**13**	**(1.1)**	**14**	**(2.1)**		27	(1.5)
**Language survey completed with**					<0.001		
Arabic	**490**	**(42.1)**	**396**	**(58.8)**		886	(48.3)
Persian	**645**	**(55.5)**	**264**	**(39.1)**		908	(49.5)
English	**19**	**(1.6)**	**6**	**(0.9)**		25	(1.4)
Urdu	**9**	**(0.8)**	**8**	**(1.2)**		17	(0.9)
**Number of days walked so far ^b^**							
1 day	**185**	**(19.3)**	**112**	**(22.3)**		297	(20.3)
2–3 days	**518**	**(54.1)**	**302**	**(60.0)**		820	(56.2)
4–7 days	**195**	**(20.4)**	**75**	**(14.9)**		270	(18.5)
8+ days	**59**	**(6.2)**	**14**	**(2.8)**		73	(5.0)
**Type of accommodation ^c^**							
Mawkib (tent/outside)	**584**	**(50.2)**	**187**	**(27.7)**	<0.001	770	(41.9)
Private house	**370**	**(31.8)**	**182**	**(27.0)**	0.029	552	(30.0)
Mosque/hussayniya	**276**	**(23.7)**	**254**	**(37.7)**	<0.001	530	(28.9)
Permanent mawkib (built structure)	242	(20.8)	165	(24.5)	0.07	407	(22.2)
Hotel	39	(3.4)	18	(2.7)	0.413	57	(3.1)
Other	90	(7.7)	62	(9.2)	0.278	152	(8.3)
**Source of food ^c^**							
Mawkib (outside cooking)	**742**	**(63.8)**	**368**	**(54.6)**	<0.001	1110	(60.4)
Mawkib (kitchen facility)	**508**	**(43.7)**	**435**	**(64.5)**	<0.001	943	(51.3)
Private home food	**137**	**(11.8)**	**94**	**(13.9)**	0.181	231	(12.6)
Street food—donated	**496**	**(42.6)**	**327**	**(48.4)**	0.016	822	(44.7)
Street food—sold	**42**	**(3.6)**	**57**	**(8.5)**	<0.001	99	(5.4)
Restaurant	22	(1.9)	14	(2.1)	0.786	36	(2.0)
Other	10	(0.9)	7	(1.0)	0.702	17	(0.9)

The bolded cells highlight statistically significant differences. ^a^ Four individuals did not have gender recorded and so are not included in this table. This table has data for 1838 out of 1842 participants. Row totals may vary because of missing numbers; e.g., 23 participants did not provide age. ^b^ Including the interview day. ^c^ more than one response is possible.

**Table 2:** Symptoms of potential respiratory infectious diseases in Arbaeen 2019 survey participants.

	Male		Female		*p*-Value	Total	
**Symptom**	***n***	**(%)**	***n***	**(%)**		***n*** **^a^**	**(%)**
**Fever**	101	(8.8)	47	(7.1)	0.202	148	(8.2)
**Respiratory symptoms**							
**Cough**	**312**	**(27.3)**	**149**	**(22.6)**	**0.029**	**461**	**(25.6)**
**DURATION (DAYS)**					0.098		
**0**–2	222	(19.1)	80	(11.9)		302	(16.4)
**3**–7	68	(5.8)	47	(7.0)		115	(6.2)
**8**–14	2	(0.2)	4	(0.6)		6	(0.33)
**15**–30	2	(0.2)	1	(0.1)		3	(0.15)
**31+**	3	(0.3)	7	(1.0)		10	(0.54)
**Sputum**					0.012		
**YES**	**155**	**(13.3)**	**55**	**(8.5)**		**210**	**(11.4)**
**NO**	**157**	**(13.5)**	**93**	**(14.4)**		**250**	**(13.6)**
**Colour of sputum ^b^**							
**WHITE**	110	(9.5)	33	(4.9)		143	(7.8)
**GREEN**	37	(3.2)	17	(2.5)		54	(2.9)
**BROWN/BLACK**	8	(0.7)	2	(0.3)		10	(0.5)
**BLOOD-STAINED**	1	(0.1)	1	(0.1)		2	(0.1)
**Breathlessness**	130	(11.4)	88	(13.5)	0.196	218	(12.1)
**Sore throat**	213	(18.8)	109	(16.6)	0.231	322	(18.0)
**Cough+ fever + sore throat**	37	(3.2)	21	(3.1)	0.210	58	(3.1)

The bolded cells highlight statistically significant differences. ^a^ Four individuals did not have gender recorded and so are not included in this table. This table has data for 1838 out of 1842 participants. Row totals may vary because there were missing numbers as follows: fever *n* = 32, cough *n* = 36, breathlessness *n* = 43, sore throat = 51. ^b^ more than one response is possible.

**Table 3:** Predictors of respiratory symptoms that developed during the walk in 1842 Arbaeen participants.

	Cough			Breathlessness			Fever			Any of These Symptoms			Total	
	*n*	(%) ^a^	*p*-Value	*n*	(%) ^a^	*p*-Value	*n*	(%) ^a^	*p*-Value	*n*	(%) ^a^	*p*-Value	*n* ^b^	(%) ^c^
**Gender (%)**			0.001			0.197			0.154			0.230		
Male	**284**	**(25.1)**		107	(9.5)		97	(8.5)		362	(32.1)		1163	(63.3)
Female	**121**	**(18.6)**		74	(11.4)		44	(6.6)		191	(29.4)		675	(36.7)
**Age (years) (%)**			<0.001			<0.001			0.296			0.002		
16–24	**136**	**(32.1)**		**70**	**(16.4)**		32	(7.4)		**165**	**(38.9)**		438	(24.1)
25–44	**169**	**(20.7)**		**75**	**(9.1)**		68	(8.2)		**237**	**(29.0)**		841	(46.2)
45–64	**91**	**(19.2)**		**26**	**(5.6)**		32	(6.7)		**133**	**(28.4)**		487	(26.7)
65+	**5**	**(9.6)**		**9**	**(17.0)**		5	(9.4)		**12**	**(23.1)**		54	(3.0)
**Recruitment station**			<0.001			0.001			0.768			<0.001		
1	**64**	**(11.3)**		**38**	**(6.7)**		44	(7.6)		**102**	**(18.2)**		560	(30.4)
2	**340**	**(28.5)**		**143**	**(12.0)**		96	(8.0)		**449**	**(37.7)**		1194	(64.8)
**No of days walked so far (%)**			<0.001			<0.001			0.001			<0.001		
**5 or fewer**	**339**	**(20.9)**		**149**	**(9.2)**		**120**	**(7.3)**		**467**	**(29.0)**		1671	(93.2)
**6+**	**66**	**(55.9)**		**32**	**(27.8)**		**21**	**(17.4)**		**86**	**(72.9)**		122	(6.8)
**Type of country of origin (%) ^d^**			0.029			0.133			0.110			0.288		
High income	**2**	**(5.4)**		5	(14.3)		0	(0)		7	(19.4)		37	(2.0)
Upper middle income	**390**	**(23.1)**		174	(10.3)		132	(7.7)		530	(31.5)		1734	(94.1)
Low to lower middle income	**11**	**(28.2)**		0	(0)		6	(14.0)		11	(28.2)		44	(2.4)
Unknown/missing	**2**	**(9.1)**		2	(8.0)		3	(12.0)		5	(20.8)		27	(1.5)
**Source of food**			0.001			0.135			0.497			<0.001		
Mawkib (outside cooking)														
Yes	**277**	**(25.4)**		120	(11.0)		82	(4.5)		**375**	**(34.4)**		1111	(60.3)
No	**128**	**(18.4)**		61	(8.8)		59	(3.3)		**178**	**(25.8)**		731	(39.7)
**Mawkib (kitchen facility)**			0.016			0.752			0.005			0.141		
Yes	**189**	**(20.4)**		95	(10.3)		**57**	**(6.1)**		273	(29.5)		945	(51.3)
No	**216**	**(25.1)**		86	(9.9)		**84**	**(9.6)**		280	(32.7)		897	(48.7)
**Home food**			0.763			0.207			0.002			0.957		
Yes	49	(21.9)		17	(7.7)		**29**	**(12.8)**		68	(30.9)		231	(12.5)
No	356	(22.8)		164	(10.5)		**112**	**(7.1)**		485	(31.1)		1611	(87.5)
**Street food—donated**			<0.001			0.512			0.797			0.001		
Yes	**141**	**(17.5)**		86	(10.6)		65	(8.0)		**217**	**(27.2)**		824	(44.7)
No	**264**	**(26.9)**		95	(9.7)		76	(7.6)		**336**	**(34.3)**		1018	(55.3)
**Street food—sold**			0.189			0.775			0.547			0.622		
Yes	27	(28.1)		9	(9.3)		6	(6.2)		32	(33.3)		99	(5.4)
No	378	(22.4)		172	(10.2)		135	(7.9)		521	(30.9)		1743	(94.6)
**Restaurant**			0.439			0.041			0.001			0.198		
Yes	5	(14.7)		**7**	**(20.6)**		**8**	**(23.5)**		14	(41.2)		36	(2.0)
No	400	(22.8)		**174**	**(9.9)**		**133**	**(7.5)**		539	(30.9)		1806	
Other													17	(0.9)
**Preventive measures**														
**Avoid street food—sold**			0.816			0.002			<0.001			<0.001		
Yes	154	(23.7)		**84**	**(13.1)**		**74**	**(11.3)**		**239**	**(37.1)**		657	(35.7)
No	251	(22.1)		**97**	**(8.5)**		**67**	**(5.8)**		**314**	**(27.6)**		1185	(64.3)
**Avoid street food—donated**			0.237			0.615			<0.001			<0.001		
Yes	69	(27.4)		23	(9.2)		**38**	**(15.0)**		**107**	**(42.8)**		254	(13.8)
No	336	(21.9)		158	(10.3)		**103**	**(6.6)**		**446**	**(29.2)**		1588	(86.2)
**Only packaged water (bottled or packed cups)**			<0.001			0.076			0.491			<0.001		
Yes	**127**	**(16.7)**		66	(8.7)		56	(7.3)		**196**	**(25.8)**		776	(42.1)
No	**278**	**(27.1)**		115	(11.2)		85	(8.1)		**357**	**(35.0)**		1066	(57.9)
**Avoid fresh fruits or salads**			<0.001			0.242			0.007			0.022		
Yes	**41**	**(14.5)**		34	(12.1)		**11**	**(3.8)**		**71**	**(25.3)**		287	(15.6)
No	**364**	**(24.2)**		147	(9.8)		**130**	**(8.5)**		**482**	**(32.2)**		1555	(84.4)
**Handwashing**			0.091			0.026			0.073			0.005		
Yes	271	(22.8)		**133**	**(11.3)**		103	(8.6)		**394**	**(33.3)**		1205	(65.4)
No	134	(22.4)		**48**	**(7.9)**		38	(6.2)		**159**	**(26.7)**		637	(34.6)
**Facemask**			<0.001			0.001			0.243			<0.001		
Yes	**157**	**(29.6)**		**73**	**(13.7)**		48	(8.9)		**217**	**(41.3)**		543	(70.5)
No	**248**	**(19.7)**		**108**	**(8.6)**		93	(7.3)		**336**	**(26.8)**		1299	(29.5)
Other														
**Up to date vaccination**			0.906			0.123			0.773			0.066		
Yes	242	(22.9)		109	(10.3)		83	(7.7)		339	(32.2)		1072	(60.6)
No	123	(20.6)		53	(8.9)		51	(8.3)		161	(27.0)		615	(34.7)
Don’t know	19	(23.5)		13	(16.0)		5	(6.2)		28	(34.6)		81	(4.6)
**Flu vaccination in the past year (%)**			0.357			0.200			<0.001			0.532		
Yes	57	(20.4)		36	(13.0)		**21**	**(7.4)**		79	(28.7)		279	(16.0)
No	327	(22.9)		140	(9.8)		**104**	**(7.2)**		436	(30.6)		1425	(81.5)
Do not know	4	(9.1)		3	(6.8)		**15**	**(34.1)**		21	(47.7)		44	(2.5)
**Other vaccinations for this trip**			0.705			0.032			<0.001			0.294		
Yes	22	(25.3)		**3**	**(3.4)**		**19**	**(21.8)**		23	(26.7)		86	(5.0)
No	394	(23.5)		**175**	**(10.6)**		**117**	**(7.0)**		528	(32.2)		1644	(95.0)
Missing													80	
**Regular smoking**			0.025			0.002			0.447			0.163		
Yes	**86**	**(26.3)**		**49**	**(14.9)**		23	(6.9)		111	(33.9)		327	(18.8)
No	**302**	**(21.2)**		**130**	**(9.1)**		117	(8.1)		425	(30.0)		1417	(81.3)
**Total (%)**	405	(22.7)		181	(10.1)		141	(7.9)		553	(31.06)			

The bolded cells highlight statistically significant differences. ^a^ In all columns except for the last one, row percentages are shown. These indicate the percentage who had that symptom out of the number of participants who answered that question. ^b^ Totals represent participant numbers regardless of whether they had symptoms. Row totals may vary due to missing values. ^c^ This column shows column percentages. ^d^ Country of origin was coded according to the country’s income level using the World Bank classification.

**Table 4:** Logistic regression analysis of predictors for the development of cough and any of cough, breathlessness, or fever in participants of the 2019 Arbaeen procession.

Dependent Variable	Predictors ^a^	*p*-Value ^b^ Bivariate	OR	95% CI for OR		*p*-Value ^b^ Multivariate	AOR	95% CI for AOR	
				Lower	Upper			Lower	Upper
Cough									
	Age	0.392	1.00	0.99	1.00	0.009	0.99	0.98	1.00
	Walked for 6+ days vs. walked for ≤5 days	<0.001	4.72	3.23	6.89	<0.001	4.59	3.01	7.00
	Use of facemask as a preventive measure vs. not using this measure	<0.001	1.86	1.49	2.32	<0.001	2.71	2.08	3.53
	Use of packaged water only as a preventive measure vs. not using this measure	<0.001	0.57	0.46	0.72	<0.001	0.60	0.45	0.78
	Avoiding fresh fruits/salads as a preventive measure vs. not using this measure	<0.001	0.54	0.39	0.76	0.007	0.58	0.39	0.86
	Eating mawkib food (indoor kitchen facility) vs. not using this food source	0.003	0.73	0.60	0.90	0.014	0.72	0.56	0.94
	Eating donated street food vs. not using this food source	<0.001	0.60	0.49	0.75	0.001	0.66	0.51	0.85
Any of cough, fever, or breathlessness									
	Walked for 6+ days vs. walked for ≤5 days	<0.001	6.60	4.33	10.04	<0.001	7.20	4.64	11.20
	Eating mawkib food (outside cooking) vs. not using this food source	<0.001	1.51	1.22	1.86	0.016	1.33	1.05	1.68
	Use of facemask as a preventive measure vs. not using this measure	<0.001	1.92	1.55	2.38	<0.001	2.47	1.95	3.13
	Use of packaged water only as a preventive measure vs. not using this measure	<0.001	0.65	0.53	0.80	<0.001	0.64	0.50	0.81
	Eating donated street food vs. not using this food source	0.001	0.71	0.58	0.88	0.013	0.76	0.61	0.94
	Avoiding sold street food vs. not using this preventive measure	<0.001	1.54	1.26	1.90	0.007	1.37	1.09	1.72

^a^ Only gender, age and independent variables, which were significant predictors in the multivariate model are included in this table. ^b^ Degrees of freedom = 1 in all rows.

## Data Availability

The data presented in this study are available on request from the corresponding author. The data are not publicly available due to sensitivities in that geographic region.

## Appendix A. English Version of the 2019 Arbaeen Full Survey

Note: The article focuses on responses to items relevant to respiratory symptoms. Findings relating to other items of this survey (e.g., gastrointestinal questions) will be discussed in future reports.

**The 2019 Arbaeen Survey**

**Table:**

*Before approaching participant, research assistant to:* ***1.*** ***press for automatic entry of:* Date and time** **2.** **Record interview site:** ☐ Station 1 ☐ Station 2 **3.** **Record gender of person approached:** ☐ Male ☐ Female

**Recruitment script**

*We are from the University of Kufa/Sydney. We are conducting a health survey of Arbaeen participants. This is to find out how we can help future Arbaeen participants to stay in the best health possible, during and after the Arbaeen. If you take part, your answers are confidential. We do not need your name. You can withdraw from this study at any time.*

☐ **Do you give consent to take part in this survey?**

**Yes → Skip to question 1.**

**No →**

  **If no: record:**

- **Estimated broad age group**
  ☐ less than 35
  ☐ 35–60
  ☐ More than 60
  ☐ unknown
- **Reason for refusal (stated or observed):**
  ☐ Language barrier
  ☐ Cultural reasons
  ☐ Too tired
  ☐ No time
  ☐ Suspicious about the survey
  ☐ No reason given or observed
  ☐ Other

**1. What is your age? _____**

**2. Country of residence**

☐ Iraq
  If Iraq: Town/region of residence________________ (select from dropdown list)
  ☐ Anbar
  ☐ Arbil
  ☐ Babel
  ☐ Baghdad
  ☐ Basra
  ☐ Dahuk
  ☐ Dhi Qar
  ☐ Diyala
  ☐ Karbala
  ☐ Kirkuk
  ☐ Maysan
  ☐ Muthanna
  ☐ Najaf
  ☐ Ninawa
  ☐ Qadisiyyah
  ☐ Salah ad-Din
  ☐ Sulaymaniyyah
  ☐ Wasit
  ☐ Other (please specify) ___________
  Then skip to Q4
  ☐ Iran
  ☐ Afghanistan
  ☐ Algeria
  ☐ Australia
  ☐ Austria
  ☐ Azerbaijan
  ☐ Bahrain
  ☐ Bangladesh
  ☐ Belgium
  ☐ Canada
  ☐ China
  ☐ Denmark
  ☐ Egypt
  ☐ France
  ☐ Germany
  ☐ Greece
  ☐ India
  ☐ Indonesia
  ☐ Ireland
  ☐ Italy
  ☐ Japan
  ☐ Jordon
  ☐ Kenya
  ☐ Kuwait
  ☐ Lebanon
  ☐ Malaysia
  ☐ Morrocco
  ☐ Neatherlands
  ☐ New Zealand
  ☐ Nigeria
  ☐ Norway
  ☐ Oman
  ☐ Pakistan
  ☐ Qatar
  ☐ Saudi Arabia
  ☐ Singapore
  ☐ South Africa
  ☐ Spain
  ☐ Sudan
  ☐ Sweden
  ☐ Switzerland
  ☐ Syria
  ☐ Tunesia
  ☐ Turkey
  ☐ UAE
  ☐ UK
  ☐ USA
  ☐ Yemen
  ☐ Other

**3. How many complete days have you passed in Iraq since you arrived? __________**

**4. How many days ago did you start your Arbaeen walk? (not counting today) _______________**

**5. Where did you start the walk?**

☐ Home
☐ Shrine of Imam Ali
☐ Outskirts of Najaf
☐ Stick number ……….
☐ ☐ Other

**6. Where have you stayed since you started the Arbaeen walk? (Select any that apply)**

☐ Hotel
☐ Private House
☐ Mawkib (tent/outside)
☐ Permanent Mawkib (built structure)
☐ Mosque/Hussayniya
☐ Other

**7. Where have you obtained food from since you started the Arbaeen? (Select any that apply)**

☐ Restaurant
☐ Street food (sold)
☐ Street food (donated)
☐ Home food
☐ Mawkib (outside cooking)
☐ Mawkib (kitchen facility)
☐ Other

**8. Which of the following have you paid for during the Arbaeen walk?**

☐ Accommodation
☐ Food
☐ Neither

**9. Where did you stay last night?**

☐ Hotel
☐ Private house
☐ Mawkib (tent/outside)
☐ Permanent mawkib (building structure)
☐ Mosque/hussayniya
☐ Own home
☐ Other

**9.1** Thinking of the place where you stayed last night… **(a) Did it have any toilet facility?**

☐ Yes ☐ No If no, skip to Question 10

**9.1 (b) Which of the following was there in the toilet area? (Select all that apply)**

☐ a hose
☐ a bucket of water
☐ running water for hand washing
☐ soap or detergent for handwashing
☐ a shared towel for drying hands
☐ none of the above

**10. What was the source of your last meal? (Select all that apply)**

☐ Restaurant
☐ Street food (sold)
☐ Street food (donated)
☐ Home food
☐ Mawkib (outside cooking)
☐ Mawkib (kitchen facility)
☐ Other

**10.1**. Thinking of that last place where you had a meal:

**10a. Were the food preparers wearing gloves?**

    ☐ Yes ☐No ☐ I don’t know/I could not see

**10b. Was there running water available for the food preparers/distributors?**

    ☐ Yes ☐No ☐ I don’t know/I could not see

**11. Have you had tea or coffee on the road during the walk?**

☐ Yes ☐No

If no, skip to Question 12

If yes, **what type of cup did you use?**

☐ Disposable cup
☐ Reuseable cup (given to you by others)
☐ Reuseable cup (your own)

**12. What preventative measures are you taking during the Arbaeen walk? (Select any that apply)**

☐ No street food (sold)
  ☐ No street food (donated)
☐ Only bottled water (Note: translations into the other languages had this response option as ‘bottled or packaged water’)
☐ No ice
☐ Avoiding fresh fruits or salads
☐ Carrying antibiotics
☐ Carrying medicine to stop diarrhoea
☐ Hand washing
☐ Face mask
☐ Other

**13a. Since you started your journey, have you experienced *coughing?***

    ☐ Yes ☐ No (if no, skip to question 12b)

    (If yes) **Are you coughing up phlegm/sputum)?** ☐ Yes ☐ No (if no, skip to duration)

        If yes, Colour of phlegm (Select any that apply)

        ☐ white ☐green ☐ brown/black ☐ blood stained

    Duration _______________days

**13b. Since you started your journey, have you experienced *breathlessness?***

    ☐ Yes ☐ No (if no, skip to question 13c)

    (If experienced)

    **When do you get short of breath?**

    ☐ when standing still ☐walking on flat ground ☐ walking up hills/steps

    ***(Select any that apply)***

    Duration? (days) ________________

    Does/did anything make it better? (Select any that apply)

☐ Inhaler, like Ventolin (salbutamol)
☐ Heart tablet (like nitroglycerin)
☐ Antibiotics
☐ Other
☐ No, nothing makes it better

**13c. Since you started your journey, have you had a *sore throat?***

☐ Yes ☐ No

**13d. Since you started your journey, have you experienced *fever?***

☐ Yes ☐ No

(If yes) Fever duration? ________________ days

*---> if fever experienced* **When you had a fever did you also have a cough and sore throat?**

    ☐ Yes ☐No

**13e.** *(If any of the symptoms in Q12 were experienced):*

**Have you received treatments for any of the above conditions?**

☐ Yes ☐ No If no, skip to Q

    If yes, Type of treatment received:

        ☐ symptom relief

*---> (If yes)* in which form were they given?

        ☐ oral (by mouth) ☐ injectable ☐ inhaler

*---> (If injectable)*, What type of needle was used for the injection?

        ☐ a single use needle ☐ a reusable needle ☐ Don’t know

        ☐ antibiotics

*---> (If yes)* in which form were they given?

        ☐ oral (by month) ☐ injectable

        *---> (If injectable)* What type of needle was used for the injection?

        ☐ a single use needle ☐ a reusable needle ☐ Don’t know

        ☐ traditional medicine

        ☐ other

        Who advised you to take this medication? (select all that apply)

        ☐ self ☐family/friends ☐pharmacist ☐ doctor ☐ other

**14. Now I’m going to ask you about gut symptoms.**

**14a. Have you experienced diarrhoea during your journey?**

☐ No ☐Yes, diarrhoea ☐Yes, vomiting

*---> (if yes)* Number of motions per day: ☐ 2 or less ☐3 or more

    Duration ________________

    Did your stools have..? (Select any that apply)

    ☐ blood ☐ mucus ☐ neither

    When you had diarrhoea, did you have fever at the same time?

    ☐ Yes ☐ No

    *---> (If vomiting experienced)* Number of times/day _____________

    Duration _________________

***14b. If vomiting or diarrhoea were experienced:***

**Did you take any treatments for diarrhoea or vomiting?**

    ☐ Yes ☐ No (if no, skip to Q14)

    If yes, Type of treatment received (Select all that apply)

        ☐ tablet to stop diarrhoea

        ☐ rehydration fluids

        ☐ antibiotics

        ☐ traditional medicine

        ☐ intravenous fluids

        ☐ other

        Who advised you to take this medication? (select all that apply)

        ☐ self ☐family/friends ☐pharmacist ☐ doctor ☐ other

**15. Have you been injured during the Arbaeen walk?**

    ☐ Yes ☐ No

    If yes, Was your injury a…? (select all that apply)

    ☐ cut ☐fall ☐broken bone ☐ sprained ankle ☐ car accident ☐ other

**16. Have you experienced any other health problems during the Arbaeen walk?**

    ☐ Yes ☐ No (if no, skip to Q 16)

      If yes, What type of health problems were they (in broad terms)? (Select any that apply)

☐ Chest pain
☐ Heart problem
☐ Diabetes
☐ Neurological condition (e.g., stroke)
☐ Other

**17. Did you seek any health care during the Arbaeen?**

    ☐Yes

    ☐ No (If no, skip to Q17)

    (If yes) 17a. For what condition? (Select any that apply)

☐ Chest pain
☐ Heart problem
☐ Diabetes
☐ Neurological condition (e.g., stroke)
☐ Other

    17b. What treatment did you receive? (Select any that apply)

☐ Nothing
☐ Antibiotics
☐ Pain killer
☐ Other medicine(s)
☐ Oxygen
☐ Hospitalisation
☐ Surgery
☐ Other

**18. Do you have any pre-existing conditions? (Select any that apply)**

☐ No
☐ Heart problems
☐ Lung problems, including asthma
☐ Diabetes
☐ Kidney disease
☐ Bowel disease
☐ Neurological conditions (eg. stroke)
☐ Hypertension
☐ Other

**19. For women: Is it possible that you are pregnant?**

☐ No ☐ Yes ☐ Unsure ☐ Don’t want to answer

**20. Do you think you are up to date with your vaccinations?**

    ☐ No ☐ Yes ☐ I don’t know

**21. Have you had an influenza (flu) vaccination in the past year?**

    ☐ No ☐ Yes ☐ I don’t know

**22. Did you receive any vaccination specifically for this trip? (other than the flu vaccination)**

☐ No ☐ Yes

**23. Before the Arbaeen, did you usually smoke most days?**

☐ No ☐ Yes
